# Grain size dependent high-pressure elastic properties of ultrafine micro/nanocrystalline grossular

**DOI:** 10.1038/s41598-021-01960-6

**Published:** 2021-11-18

**Authors:** Jin S. Zhang, T. Irifune, M. Hao, D. Zhang, Y. Hu, S. Tkachev, P. Dera, J. Chen, Ying-Bing Jiang, Adrian J. Brearley, J. D. Bass, V. Prakapenka

**Affiliations:** 1 Institute of Meteoritics, University of New Mexico, Albuquerque, NM 87131 USA; 2 Department of Earth and Planetary Sciences, University of New Mexico, Albuquerque, NM 87131 USA; 3 Geodynamics Research Center (GRC), Ehime University, Matsuyama, Ehime 790-8577 Japan; 4 Center of Advanced Radiation Sources, University of Chicago, Chicago, IL 60637 USA; 5 Department of Geology and Geophysics, School of Ocean and Earth Science and Technology, Hawaii Institute of Geophysics and Planetology, University of Hawaii at Manoa, Honolulu, HI 96822 USA; 6 School of Earth and Space Sciences, University of Science and Technology of China, Hefei, 230026 China; 7 Department of Geology, University of Illinois, Urbana, 61801 USA

**Keywords:** Mineralogy, Nanoscale materials

## Abstract

We have performed sound velocity and unit cell volume measurements of three synthetic, ultrafine micro/nanocrystalline grossular samples up to 50 GPa using Brillouin spectroscopy and synchrotron X-ray diffraction. The samples are characterized by average grain sizes of 90 nm, 93 nm and 179 nm (hereinafter referred to as samples Gr90, Gr93, and Gr179, respectively). The experimentally determined sound velocities and elastic properties of Gr179 sample are comparable with previous measurements, but slightly higher than those of Gr90 and Gr93 under ambient conditions. However, the differences diminish with increasing pressure, and the velocity crossover eventually takes place at approximately 20–30 GPa. The X-ray diffraction peaks of the ultrafine micro/nanocrystalline grossular samples significantly broaden between 15–40 GPa, especially for Gr179. The velocity or elasticity crossover observed at pressures over 30 GPa might be explained by different grain size reduction and/or inhomogeneous strain within the individual grains for the three grossular samples, which is supported by both the pressure-induced peak broadening observed in the X-ray diffraction experiments and transmission electron microscopy observations. The elastic behavior of ultrafine micro/nanocrystalline silicates, in this case, grossular, is both grain size and pressure dependent.

## Introduction

Mg-Fe-Ca bearing garnet with approximate composition (Mg, Fe, Ca)_3_Al_2_Si_3_O_12_, is one of the major minerals in Earth’s upper mantle^1^. At depths greater than ~ 300 km depth, pyroxenes start to dissolve into the garnet structure, changing the composition of upper mantle garnet toward Mg, Ca, and Si-rich^2,3^. Assuming a pyrolitic upper mantle composition, the overall content of garnet thus increases from ~ 15% in the uppermost mantle to ~ 45% in the transition zone, and then gradually decreases due to the garnet to bridgmanite and CaSiO_3_ perovskite phase transition at 500–700 km depth^4^. Moreover, subducted oceanic crust transforms into garnetite in the transition zone^3^. Therefore, determination of the high pressure–temperature elastic properties of garnet is important for understanding the upper mantle seismic structures and subduction dynamics in the Earth’s interior.

Accurate determination of the elastic properties of natural upper mantle garnets relies on the complete characterization of the high pressure–temperature elastic properties of the relevant end-member garnets, which are pyrope Mg_3_Al_2_Si_3_O_12_, almandine Fe_3_Al_2_Si_3_O_12_, grossular Ca_3_Al_2_Si_3_O_12_ and majorite MgSiO_3_. Since the 1960s, numerous high-pressure high-temperature elasticity measurements and theoretical investigations have been made on natural and synthetic garnets with different chemical compositions^5–19^. However, near Ca-Al end-member grossular samples are less studied^5,16–18^. Previous high-pressure sound velocity measurements of end-member grossular samples include a single-crystal Brillouin spectroscopy study up to 10 GPa at 300 K^5^, an ambient temperature micro-polycrystalline ultrasonic study up to 3 GPa^16^, as well as two recent in-situ micro-polycrystalline ultrasonic studies combined with X-ray diffraction at simultaneous high pressure–temperature conditions^17,18^. Among all these studies, only Kono et al. have covered the entire pressure range of grossular’s stability field in the Earth’s interior^17^. In addition, the transition from garnet to the lower mantle mineral phases (including CaSiO_3_ perovskite and bridgmanite) is kinetically sluggish and spreads over a wide depth range^4^. Thus, within the relatively cold regions inside the Earth, garnet stability may exceed the upper mantle and transition zone and extends into the lower mantle, affecting the density contrast between the ambient mantle and the garnet-rich subducted slab crusts^2^. Therefore, exploring the density and elasticity evolution of garnets in the pressure–temperature space beyond its thermodynamic stability field (> 30 GPa) is important for understanding the dynamic instability of the subducting slabs in the deep Earth.

The elastic properties of polycrystalline materials can be estimated from the elasticity tensors of their single-crystal counterparts, and this is the approach commonly used for studying the elasticity of mantle silicates under deep Earth conditions^5,7–9,12,13^. The grain sizes of the minerals in the Earth’s mantle are approximately mm to cm in scale and the seismic wave frequencies are typically on the order of 0.1 to 10 Hz. Laboratory experiments, on the other hand, are measuring the sound velocities of polycrystalline samples with micron to submicron grain sizes at GHz to MHz frequency range^13–18^. Although the body wave dispersion in terms of frequency is very small, applying laboratory sound velocity measurements toward real Earth problems requires good understanding of the grain size effect on the elastic properties of mantle minerals at elevated pressure and/or temperature conditions^20^. As a frequently observed phenomenon in the material science community, the elastic and plastic properties of ultrafine micro/nanocrystalline materials can deviate from their microcrystalline or single-crystal counterparts beyond any theoretical bounds and experimental uncertainties^20–25^. The increase of hardness of materials with grain size reduction is known as the Hall–Petch effect, whereas the softening associated with decreasing grain size is called the inverse Hall–Petch effect^21,25^. Hardness, in many cases, is closely related to the elastic properties of materials, although they do not necessarily always couple in a direct way, especially in cases where reverse plastic deformation takes place^22^. A couple of studies suggest that the bulk modulus of nanocrystalline materials first gradually increases and then quickly decreases with grain size reduction, and the maximum bulk modulus is associated with a critical grain size^23,24^. Unfortunately, such investigations are mostly performed on metals, metal sulfides, and oxides using X-ray diffraction with static compression. Direct elasticity measurements of nanocrystalline materials at high-pressure conditions are limited (e.g. MgO^20^), and the grain size dependence of elasticity for silicates, in particular, mantle silicates remains less well understood.

Brillouin spectroscopy has been used for elasticity measurements of transparent samples, such as mantle silicates, since 1970s^26^. Its application is usually limited to transparent single crystals due to the poor optical quality of micro-polycrystalline samples resulting from the large sample grain size and/or porosity. Only a few exploratory experiments have been performed on polycrystalline lower mantle minerals up to Mbar pressure range due to the difficulties of sample synthesis and preparation^27,28^. In such cases, the signal–noise ratio is usually low, and therefore the experiments require long exposure time (up to dozens of hours). Recent developments in nanocrystalline material synthesis at ultra-high pressure–temperature conditions enable making transparent polycrystalline silicate samples suitable for Brillouin spectroscopy experiments for the first time^25,29^. The grain size of nanocrystalline silicate samples is sensitive to the pressure and temperature conditions during sample synthesis. Some of these ultrafine grained polycrystalline samples have minimal porosity and are optically as transparent as the corresponding single crystals, thus making them ideal for Brillouin spectroscopy experiments.

In this study, we report new high-pressure sound velocity and unit cell volume measurements for synthetic ultrafine micro/nanocrystalline grossular samples Gr90, Gr93, and Gr179 with 90 nm, 93 nm, and 179 nm average grain sizes, respectively, up to 50 GPa using Brillouin spectroscopy and synchrotron X-ray diffraction. This study provides the first elasticity measurements beyond the stability field of garnet in the Earth’s interior, and also the first Brillouin spectroscopy measurements for ultrafine micro/nanocrystalline silicate samples synthesized at ultra-high pressure–temperature conditions.

### Sample description and experimental methods

**Figure 1:**
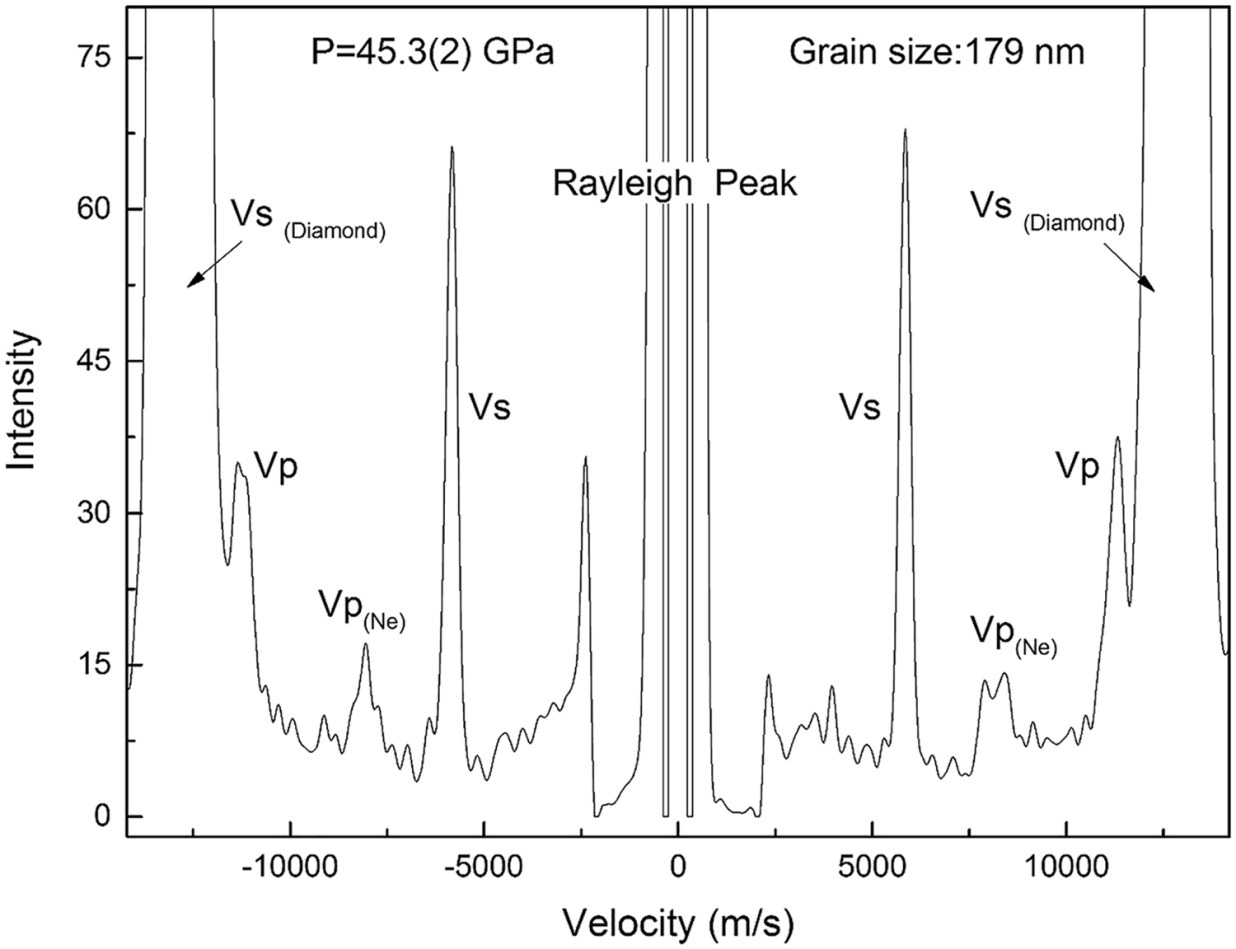
Typical Brillouin spectrum of ultrafine micro/nanocrystalline grossular. The spectrum was measured for Gr179 sample at 45.3 GPa with exposure time of 13 min.

All ultrafine micro/nanocrystalline grossular samples were synthesized using the 3000-ton multi-anvil press (ORANGE-3000) at the Geodynamics Research Center, Ehime University. The three samples (Gr90, Gr93, Gr179) with averaged grain sizes 90 nm +/− 36 nm, 93 nm +/− 54 nm, and 179 nm +/− 58 nm were synthesized at 15 GPa, 1400 °C, 1500 °C, and 1600 °C, respectively. A field emission scanning electron microscope (SEM, JEOL JSM-7000F) with an energy-dispersive X-ray spectrometer (Oxford X-Max 20), a focused ion beam system (FIB, JEOL JEM-9310FIB) system, and a transmission microscope (TEM, JEOL JEM-2100F) operated at 200 kV, were used for microstructural, grain size and chemical composition analyses for the samples before the Brillouin and X-ray diffraction experiments. The results indicate that the glass starting material and the synthetic ultrafine micro/nanocrystalline grossular samples all have identical bulk compositions of Ca_3_Al_2_Si_3_O_12_ within the uncertainties of the measurements (< 0.5%). The detailed sample synthesis and characterization procedures have been reported previously^29^. All three samples were double-side polished into pellets with 8–12 µm thickness using Al_2_O_3_ or diamond abrasive film (down to 0.3 µm grain size). All samples were scratch-free under optical examination and cut into 15–40 µm-wide pieces for diamond anvil cell (DAC) experiments. They show excellent optical quality and the Brillouin spectra collected at all pressures have high signal-to-noise ratios (Figs. 1, S1).

We have performed both Brillouin spectroscopy and synchrotron X-ray diffraction experiments on all three samples up to 50 GPa at ambient temperature. Symmetric DACs with tungsten carbide backing seats were used for generating high pressures. For synchrotron X-ray diffraction experiments, all three samples ~ 20 μm in size were loaded together in the same DAC sample chamber with two ruby spheres (Fig. S1). For Brillouin spectroscopy measurements, the samples are loaded separately into different DACs with at least two ruby spheres. The samples used for Brillouin spectroscopy measurements were slightly larger, about 40 μm in size. The diameter of the diamond cutlet is 350 µm. Pre-indented Re foils with 45–50 µm thickness are used as gaskets. The pressure-transmitting medium Ne was gas-loaded into the DAC sample chamber using the GeoSoilEnviroCARS (GSECARS) gas-loading system at the Advanced Photon Source^30^.

High-pressure X-ray diffraction experiments were carried out at the experimental stations 13-BM-C and 13-BM-D of GSECARS^31^. At 13-BM-C, the monochromatic X-ray beam is operated at 28.6 keV with 1 eV bandwidth. A Kirkpatrick-Baez mirror system is used to obtain a vertical × horizontal focus spot size of 20 μm × 15 μm, measured as full width at half maximum (FWHM). The MAR165 Charge Coupled Device (CCD) detector (Rayonix) is placed about 160 mm away from the sample on a rotational detector arm. At 13BMD, the monochromatic X-ray beam operates at 37 keV, and a stationary Perkin-Elmer image plate is used as the area detector. LaB_6_ powder is used to calibrate the distance and tilting of the detectors. Membrane pressure controllers are used to adjust the pressure remotely, and pressures are determined from the equation of state of Ne with ruby fluorescence as a secondary standard before and after each diffraction experiment^32,33^.

The Brillouin spectroscopy experiments were performed at the high-pressure laser spectroscopy laboratory at University of New Mexico (Fig. S3, Ref.^34^). A 532 nm diode-pumped solid-state laser is used as an excitation source at a constant output power of 300 mW, and approximately 50 to 60% of the power reaches the sample due to the absorption and/or reflection from the optical elements in the optical path. Pressures are determined by ruby fluorescence measured by the HR2000 + Ocean Optics spectrometer before and after the experiment. We use 50° forward symmetric scattering geometry due to the limited 60° optical opening of the DAC. Under symmetric scattering geometry, the measured velocities are independent of the refractive index of the measured material^35^. The scattering angle is calibrated using a standard silica glass 7980 (Corning Inc.) which has been precisely measured using GHz ultrasonic interferometry^36^. The scattering angle is calibrated to be 50.37(5)°, and the uncertainty of 0.05° propagates to a velocity uncertainty of ~ 0.1%, which is within the ~ 20 m/s resolution limit of Brillouin spectroscopy. The two single-crystal diamond anvils are orientated in such a way that the fast and slow directions match each other. For each sample, P-wave (Vp) and S-wave velocities (Vs) are measured at a minimum of 4 different directions on both sides to correct any possible geometrical errors introduced by the non-parallelism between the sample and the two diamond anvils. A typical Brillouin spectrum at 45.3(2) GPa is shown in Fig. 1. One compressional and one shear mode of the sample associated with the compressional mode of Ne are usually observed. At pressures close to 15 GPa, the compressional mode of Ne overlaps with the shear mode of the grossular sample, thus individual measurements of pure Ne are made to differentiate them (Fig. S2).

The recovered samples after the high-pressure Brillouin spectroscopy and X-ray diffraction experiments are further analyzed using FIB and TEM at the University of New Mexico. FIB thin foils of the grossular samples were prepared using a FEI Quanta 3D Dual beam Field Emission Gun SEM/FIB instrument at UNM. A platinum strip, 15 µm long, 2 µm wide and 2 µm thick was first deposited over the area of interest to protect the sample from ion beam damage. The FIB thin foil preparation was carried out at an ion beam accelerating voltage of 30 kV using beam currents ranging from 1 to 5 nA. An Omniprobe 200 micromanipulator was used to remove the thin foils from the thin section using the in situ lift out technique. After mounting the foils on copper TEM half grids, the samples were milled to electron transparency also using an ion beam accelerating voltage of 30 kV, with beam currents decreasing from 0.5 nA to 50 pA at the final stage of ion thinning. TEM observations were made at a JEOL NEOARM Aberration Corrected Field Emission Gun Scanning Transmission Electron Microscope operating at 200 kV. A variety of different TEM techniques were used to study the sample including bright-field TEM, high-angle annular dark-field STEM, and selected area electron diffraction. In-situ X-ray analyses were obtained with twin JEM 100 mm^2^ SDD detectors controlled by an Oxford Instruments AZtec EDS X-ray analysis system. The EDS analyses were obtained at an accelerating voltage of 200 kV.

## Results and discussion

**Figure 2:**
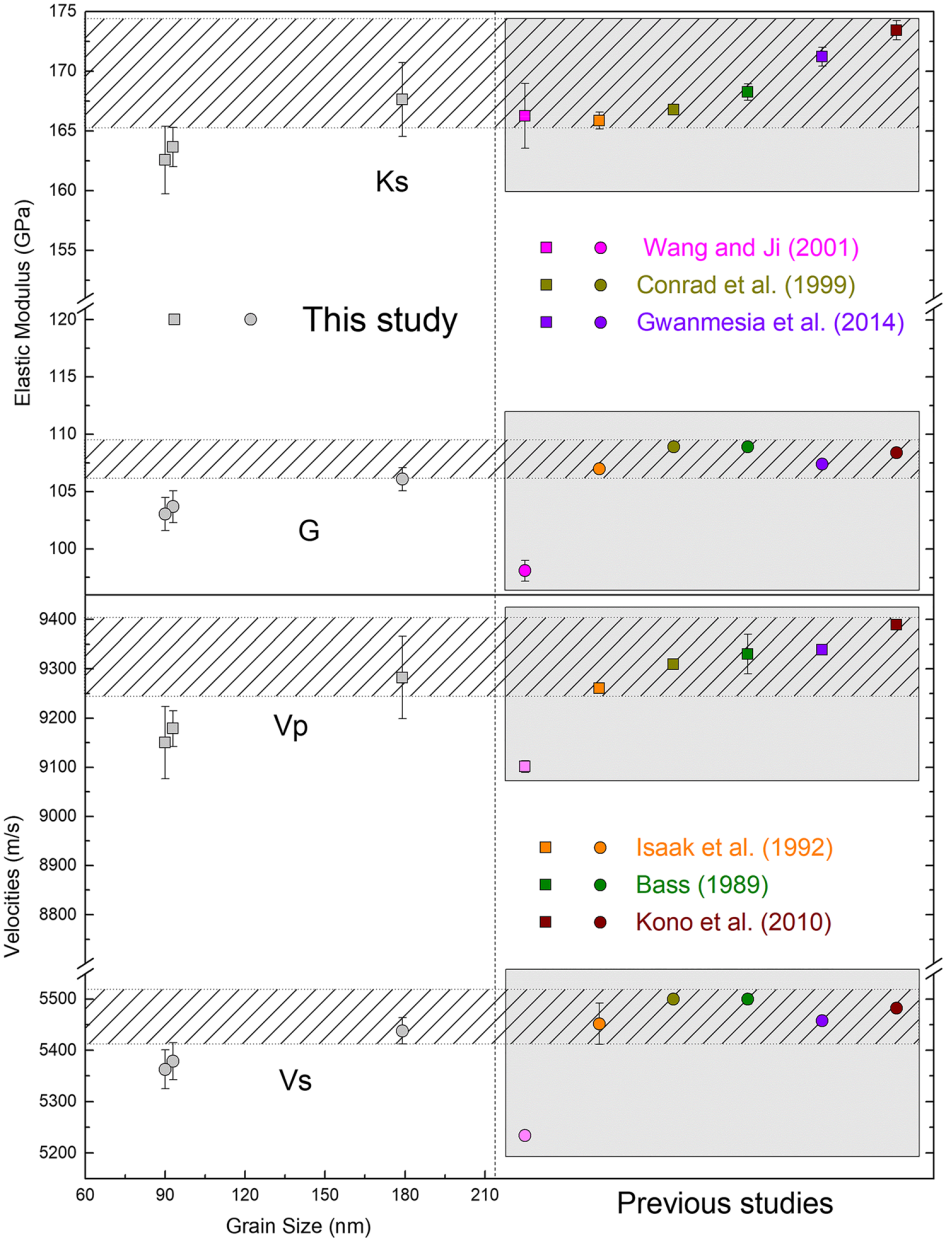
Elastic properties of ultrafine micro/nanocrystalline grossular samples at ambient condition. Previous single-crystal and micro-polycrystalline studies are also included for comparison.

At ambient conditions, the velocities measured for the three ultrafine micro/nanocrystalline samples are equal or 1–1.5% smaller than previous measurements of near-end-member grossular samples (Gr > 97%)^5,17–19,37^, except the study by Wang et al.^16^, shown as the pink symbols in Fig. 2. The lower values are probably caused by the sample porosity due to the probably incomplete sintering process and the relatively small measurement pressure range (~ 3 GPa), which are in agreement with the higher Ks’ and G’ values. It is also worth noting that, although the Vp and Vs of all three ultrafine micro/nanocrystalline samples are similar, a systematic increase of the velocities with grain size is observed (Fig. 2). The velocities of the Gr179 sample agree well with previous values obtained from either the direct measurements of micro-polycrystalline samples^17,18^ or from the Voigt-Reuss-Hill average of the experimentally determined single-crystal elasticity tensors^5,19,37^.

**Figure 3:**
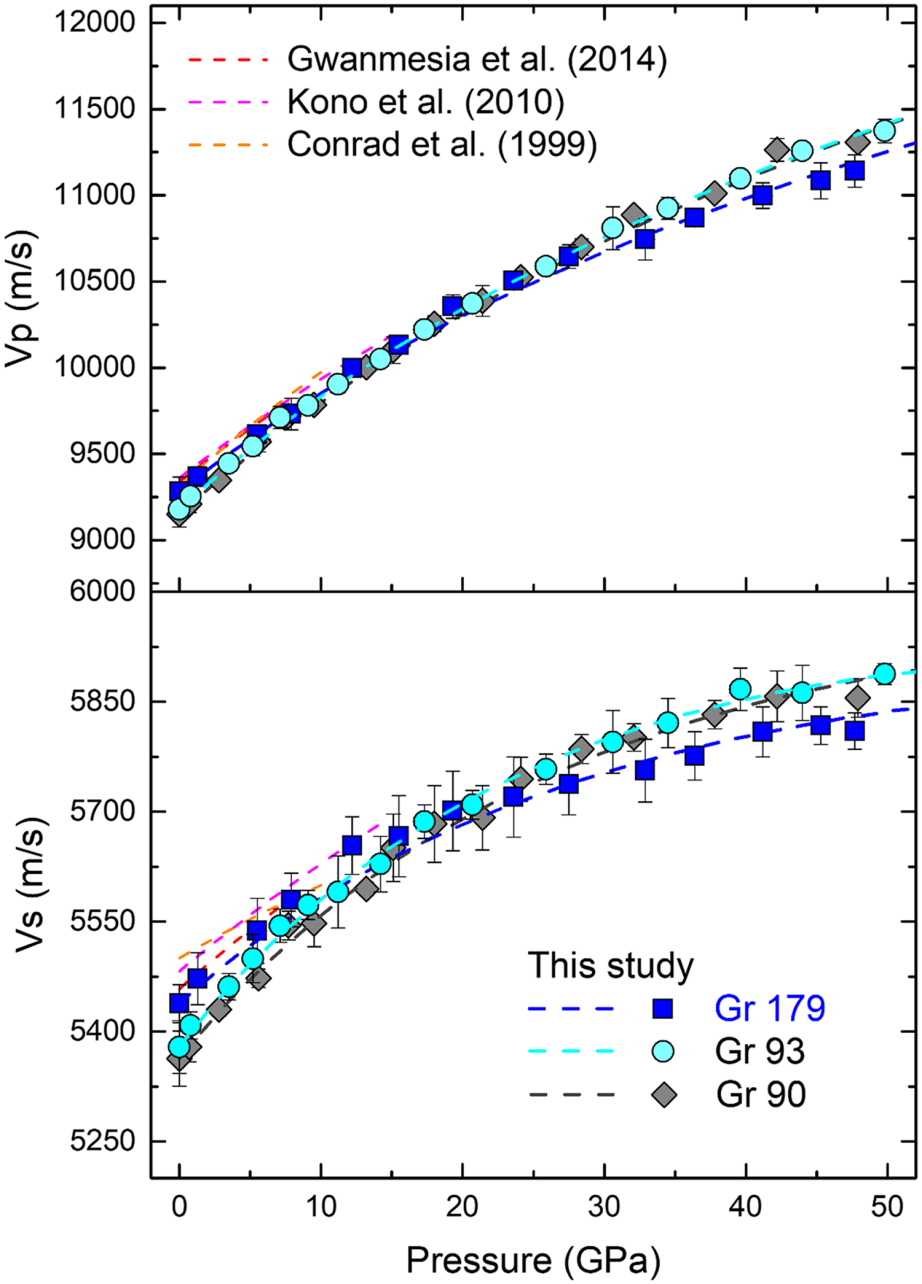
Sound velocities of ultrafine micro/nanocrystalline grossular up to 50 GPa. Previous studies are plotted with dashed lines in red, magenta and orange colors.

The P-Vp-Vs data set is then used to fit a set of finite strain equations of state to obtain the pressure derivatives of the adiabatic bulk and shear modulus for all three samples^38,39^. The ambient bulk (Ks_0_) and shear moduli (G_0_) are fixed to avoid the large trade-offs between the ambient pressure values and their pressure derivatives Ks’ and G’. Because of the pronounced curvature in the trends of the velocities, especially Vs (Figs. 3, S2), a 4th order finite strain equation of state is used. The results are summarized in Table 1. The Gr179 sample shows similar high-pressure elastic properties compared with previous single-crystal and micro-polycrystalline studies, whereas the Gr90 and Gr93 samples have lower Ks_0_ and G_0_, but slightly higher Ks’ and G’. This results in the Vp and Vs crossovers between the Gr90, Gr93 and Gr179 samples at pressures between 20–30GPa. The absolute velocities of the sample Gr179 measured in this study are consistent with two previous ultrasonic studies within experimental error at pressures < 20 GPa (Fig. 3)^17,18^. It is also worth noting that a high K’ value of 5.46 and a low G’ value of 1.1 have been reported for near grossular endmember composition single-crystal garnet^5^, which can be partially explained by the lack of measurements at ambient conditions. The Ks_0_, G_0_, Ks’ and G’ values in Ref.^5^ are estimated based on the data measured at high-pressure conditions. The relatively large trade-offs between Ks_0_, G_0,_ and Ks’ G’ likely lead to overestimated G_0_ and Ks’ and underestimated Ks_0_ and G’, as shown in Table 1. For the same reason, the Vs determined in Ref.^5^ at ambient pressure is slower but converges with the Vs of Gr179 at higher pressures, most likely due to the underestimated G’. On the other hand, Vp is higher than all the other studies at the highest measured pressure (10 GPa), which can be explained by the overestimated Ks’ (Fig. 3). The inconsistencies between previous studies and the measurements of Gr179 in this study can be explained by either different experimental pressure range (e.g. < 10 GPa in Refs.^5,]^^18^; < 20 GPa in Ref.^17^), or trade-offs between the Ks_0_ and G_0_ and their pressure derivatives.

**Table 1:** High-pressure elastic properties of pure and near-endmember grossular samples.

	Finite strain EOS (P–Vp–Vs)						Birch-Murnaghan EOS (P–V)	
	Ks_0_ [GPa]	Ks’	Ks’’ [GPa^−1^]	G_0_ [GPa]	G’	G’’ [GPa^−1^]	K_T0_ [GPa]	K_T_’
**This study**								
Gr90	163 (3)	4.59 (2)	− 0.034 (2)	103 (1)	1.547 (8)	− 0.037 (1)	161.2 (fix)	4.9 (1)
Gr93	164 (2)	4.64 (2)	− 0.038 (2)	104 (1)	1.593 (9)	− 0.043 (1)	162.2 (fix)	4.9 (2)
Gr179	168 (3)	4.41 (2)	− 0.045 (4)	106 (1)	1.310 (4)	− 0.030 (1)	166.5 (fix)	4.9 (2)
Gwanmesia et al. (2014)*	171.2 (8)	4.47 (2)	–	107.4 (2)	1.29 (5)	–	169.5 (2)	4.47 (1)
Kono et al. (2010)*	171.5 (8)	4.42 (7)	–	108.4 (3)	1.27 (3)	–	170.0 (8)	4.43 (7)
Wang and Ji (2001)*	166 (3)	5.9 (5)	–	98.1 (9)	1.3 (3)	–	–	–
Conrad et al. (1999)^a^	166.82	5.46	–	108.9	1.1	–	–	–
Isaak et al. (1992)^b^	167.8 (7)	–	–	107.0 (2)	–	–	–	–
Bass (1989)^a^	168.4 (7)	–	–	108.9 (4)	–	–	–	–
Gréaux et al. (2011)^c^	–	–	–	–	–	–	166 (3)	4.0 (fix)
Zhang et al. (1999)^c^	–	–	–	–	–	–	170 (4)	5.2 (6)

*Ultrasonic interferometry; ^a^Brillouin spectroscopy; ^b^Resonant ultrasound spectroscopy; ^c^Static compression.

**Figure 4:**
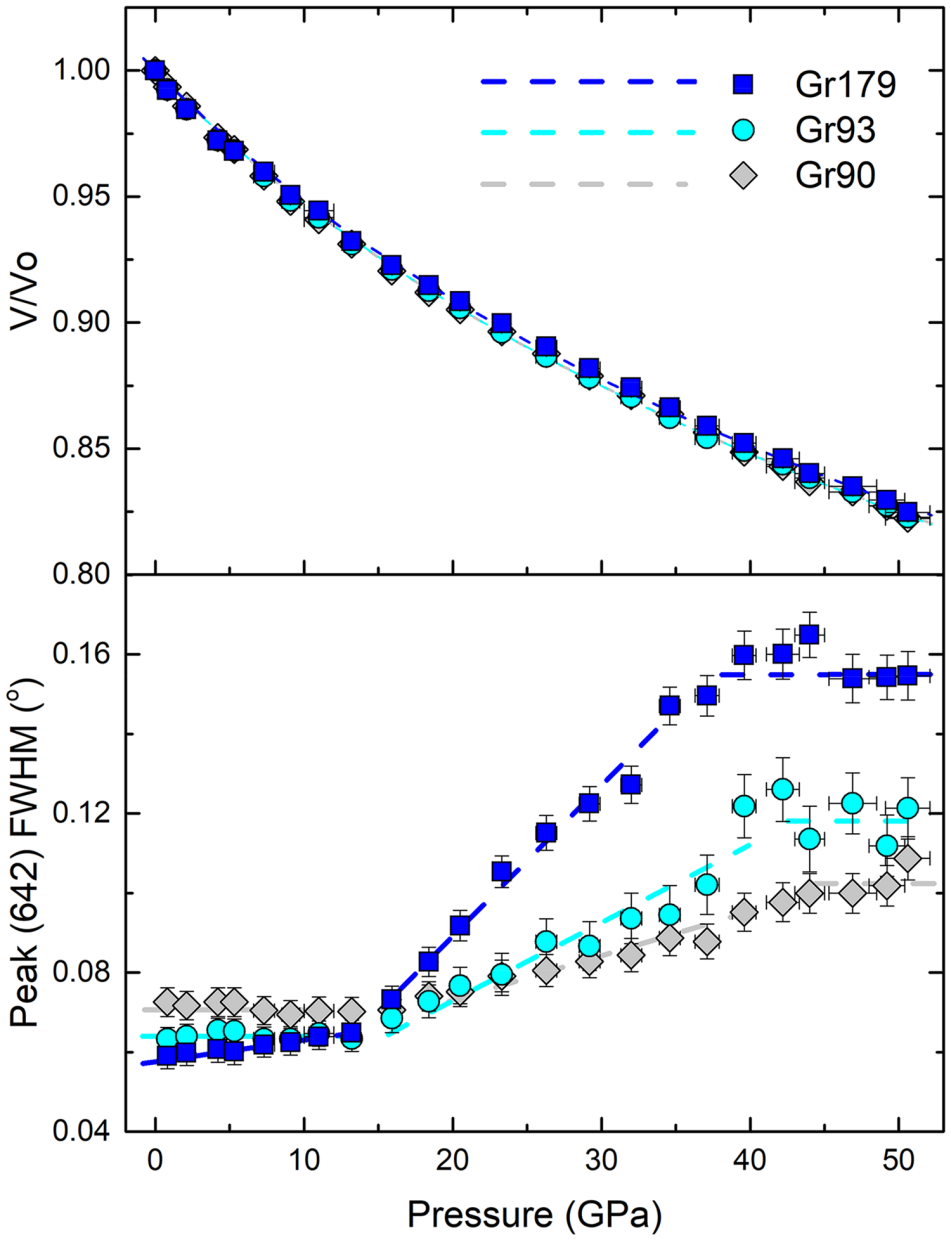
Normalized Pressure–Volume plot of ultrafine micro/nanocrystalline grossular up to 50 GPa. The FWHM of the diffraction peak (642) are shown in the bottom.

The X-ray diffraction images were converted to 1D intensity-2θ angle profiles using the Dioptas software^40^. Grossular has cubic symmetry, thus its powder diffraction pattern is very simple (Fig. S4). Software program PDIndexer is used to obtain the high-pressure unit cell parameters^41^, and 3rd order Birch-Murnaghan equation of state is fitted to the P–V data set (Fig. 4). The ambient isothermal bulk modulus is calculated from K_T0_ = Ks_0_/(1 + α*γ*T) and fixed during the fitting process. T, α and γ are temperature, thermal expansion coefficient, and Grüneisen parameter, respectively. The pressure derivatives are slightly higher, yet in agreement with the adiabatic sound velocity measurements (Table 1). We are unable to resolve the difference in K_T0_’ between the three samples based on X-ray diffraction data only.

It has been shown that the aggregate elastic properties of nanocrystalline samples can differ significantly from the usual Voigt-Reuss-Hill averaging scheme^20,23,24^. The origin of the grain size-dependent elasticity has been attributed to dislocations^42^, core–shell structures^43^, diffusion^44^, and grain boundary shearing^45^. For example, in order to explain the peaking of bulk modulus at a critical grain size for titania, Chen et al. adopted a dislocation dependant core–shell model and proposed that particles with grain sizes < 15 nm are too small to sustain the dislocations, which could lead to a significant bulk modulus reduction^23^. On the other hand, a core–shell empirical model with different elastic properties of the crystal at the center and near the surface seems to provide satisfactory explanations for the grain size dependent elasticity of PbS,TiO_2_, Cu, and Pd nanocrystalline samples^23,24,46^. It is worth noting that many of those previous studies determined the elastic properties of the loose nanometer-size particles instead of the sintered nanocrystalline aggregate materials, such as the grossular composites used in this study. The elastic properties of the loose nanoparticle powder are different from the sintered nanocomposites formed by those particles. We can use a classic spherical core–shell-interstitial ground matrix model to qualitatively describe the samples used in this study^47^. We assume the ultrafine micro/nanocrystalline grossular grains have spherical geometry and are enclosed in a boundary layer shell. The ground matrix here is the extremely small pore spaces within the sample. Due to the ultra low porosity of the samples used in this study, the volume fraction of the grossular grain plus boundary shell (c%) is thus close to 100%. Smaller grain size leads to lower porosity thus higher c values with c(Gr90) ≈ c(Gr93) > c(Gr179). The relative shell thickness tends to decrease with increasing grain size. Thus, the volume fraction of grossular grains without shells (f%) for Gr179 could be similar to or even larger than Gr90 and Gr93. As pointed by Marcadon et al., the effective elastic moduli of nanocomposites with the core–shell-interstitial ground matrix model increase with f, but decrease with c^47^. At ambient conditions, if f(Gr179) is similar to or larger than f(Gr90) and f(Gr93), then the smaller c(Gr179) can result in the higher elastic moduli of Gr179 measured in this study. In other words, increase in grain boundary area and the associated higher fraction of atoms at the grain boundaries, similar to what was described in Ref.^23^, can result in lower elastic moduli of Gr90 and Gr93 compared with Gr 179 measured at ambient conditions.

To understand the origin of the elastic modulus crossover between Gr90/Gr93 and Gr179 at high-pressure conditions, we have analyzed the diffraction peak width change as a function of pressure. Although diffraction peak broadening happens for all three samples between 15–40 GPa (Figs. 4, S4, S5), it is most significant for sample Gr179. As shown in Fig. 4, from ambient conditions to 50 GPa, the full-width-half-maximum (FWHM) of the diffraction peak (642) increases 0.1° from ~ 0.06° to ~ 0.16° for Gr 179. However, the FWHM increase of the peak (642) for Gr90 and Gr93 over the same pressure range is only ~ 0.04° to 0.05°. The pressure-induced peak broadening can be caused by the increased non-hydrostatic stress environment in the DAC sample chamber^48^, the reduced grain size of samples^20^, the gradual development of intergranular microscopic stress/strain localization^49^, or dissolution of Ne into ultrafine micro/nanocrystalline grossular samples at high-pressure conditions^50,51^. The three samples used in this study were polished down to similar thickness and loaded within the same DAC (Fig. S1), thus they are unlikely to experience significantly different non-hydrostatic stress fields. Although Ne crystallizes at ~ 4.8 GPa at 300 K, the first sign of non-hydrostaticity does not show up until 15 GPa and the pressure gradient in the DAC monotonically increases with pressure up to at least 50 GPa^52^. This gradually increased non-hydrostatic stress can contribute to the overall broadening trend of the diffraction peaks^48^ but is difficult to explain the different amount of peak broadening observed for different samples. At high-pressure conditions, especially when non-hydrostatic stresses exist in the sample chamber, inter-granular microscopic stress and lattice dislocation dominated deformation could develop within the ultrafine micro/nanocrystalline samples which may also lead to inhomogeneous strain within the sample, and eventually cause the peak broadening^49^. The gradual increase of FWHM starting from 15 GPa for all three samples coincides with the first appearance of non-hydrostaticity of the Ne pressure-transmitting medium^52^, thus the non-hydrostatic stress environment in the DAC sample chamber, which can result in inhomogeneous strain developed within the samples, may be an important contributor of the overall peak broadening observed for all three samples. Some geochemical studies suggest that noble gases may dissolve into the crystal structure of various silicates at high pressure–temperature conditions (e.g. amphiboles, serpentine, and mica)^50,51^, unfortunately, there is no evidence that such a process can take place in garnets, although the ultrafine micro/nanocrystalline grain size might make a difference. On the other hand, pressure-induced grain size reduction has been observed in polycrystalline samples (e.g. MgO^20^). Compared with the Voigt-Reuss-Hill averaged values calculated from single-crystal elasticity tensors, the sound velocities of MgO with ~ 20 nm grain size are ~ 40% lower at ambient conditions. It is worth noting that the samples used in Ref.^20^ are aggregated powders, which may have weaker grain boundary cohesion. This could cause more significant reductions in grain sizes and elastic moduli than in this study, as shown in Fig. 2. If the observed differential peak broadening is primarily induced by grain size reduction, then we would expect more significant grain size reduction of sample Gr179 during compression compared with Gr90 and Gr93, which may result in the observed velocity crossover above 30 GPa.

**Figure 5:**
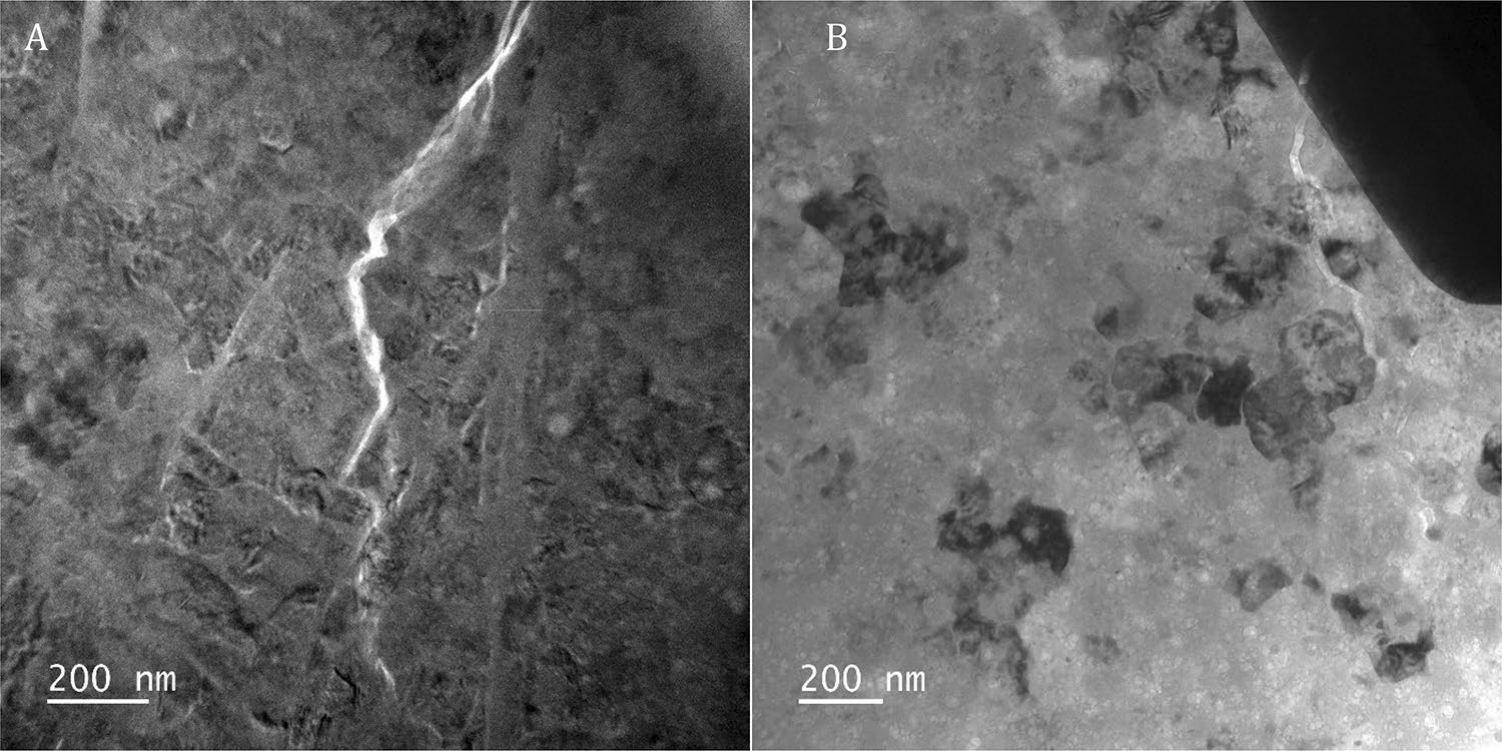
Bright-TEM images of Gr179. (**A**) TEM image of FIB prepared section showing that the samples consists of a highly-strained, dense aggregate of grossular grains with irregular shapes. The linear feature which runs N-S just to left of the center of the images appears to be a zone of shear, where significant grain size reduction has taken place, but the grossular in this zone has undergone beam damage due to electron beam irradiation; (**B**) Irregularly-shaped interlocking grossular grains in a region which appears to have undergone significant grain size reduction as indicated by a number of grains which are in strong diffracting orientations and have grains sizes < 150 nm. The grains also show significant diffraction contrast due to internal strain within the crystals. Significant regions of this area have undergone extensive electron beam induced amorphization, as indicated by the lack of diffraction contrast (all the medium gray regions). Amorphization has occurred heterogeneously within the sample, with islands consisting of crystalline grains occurring within the extensive regions of amorphized grossular.

To evaluate the two most plausible hypotheses, including the grain size reduction and strain/stress localization, we conducted TEM measurements of the ultrafine micro/nanocrystalline grossular samples Gr179 and Gr90 after quench from 50 GPa. We did not find significant grain size reduction or amorphization for Gr90. However, possible grain size reduction has been observed in Gr179. The Gr179 sample consists of anhedral, interlocking grossular crystals that exhibit very well-developed strain contrast due to internal strain (Fig. 5A and B). Although some grains have sizes of ~ 179 nm, many grains in the sample have grain sizes down to 150 nm or sometimes even less. This reduction in grain size is especially apparent in Fig. 5B, which shows a number of grains in strong diffracting orientations. However, the exact grain size reduction cannot be determined precisely, because the grossular undergoes electron beam induced amorphization very rapidly. Significant regions in Fig. 5B have undergone amorphization. It is possible that amorphization has occurred preferentially in regions of the sample that have the smallest grains size, but this could not be determined due to rapid amorphization of the sample. In Fig. 5A, a linear feature is present which appears to be a zone about 50 nm wide that represents a zone of shear. The zone has undergone complete amorphization, again, possibly due to rapid electron beam amorphization of nanometer-sized grossular grains. It seems that the inhomogeneous strain developed within the individual grains of the ultrafine microcrystalline composite Gr179 combined with the localized pressure-induced grain size reduction, are primarily responsible for the observed anomalous lower velocities and higher FWHM of Gr179 at high-pressure conditions.

## Conclusions

We have experimentally determined the high-pressure elastic properties of synthetic ultrafine micro/nanocrystalline grossular samples with grain size 90 nm, 93 nm and 179 nm using Brillouin spectroscopy and synchrotron X-ray diffraction. Three samples with various grain sizes share similar elastic properties, although systematic changes with grain size are evident. The Gr179 sample has the highest elastic moduli but the lowest pressure derivatives at ambient conditions. The Ks_0_ and G_0_ of the Gr90 and Gr93 samples are ~ 2–3% smaller than those of Gr179. The elastic properties of the Gr179 sample are comparable with previous studies. The ultrafine micro/nanocrystalline silicate samples synthesized by ultra-high-pressure methods are optically excellent for Brillouin scattering experiments, although the obtained sound velocity values need to be evaluated with caution especially for samples with smaller than 100 nm grain size. The core–shell-interstitial ground matrix model for nanocrystalline composites may provide an explanation for the grain size dependent elasticity under ambient conditions. The velocity or elasticity crossover, which happens at pressures over 30 GPa might be caused by the differential grain size reduction and/or inhomogeneous strain within the individual grains for the three different samples, which is supported by the pressure-induced peak broadening observed in the X-ray diffraction experiments and the TEM observations. Future investigations on similar ultrafine micro/nanocrystalline composites with widely different particle sizes are needed to further clarify the contribution of the grain size reduction to the observed abnormal velocity reductions at high-pressure conditions.

## Supplementary Information

Supplementary Information.

## Data Availability

All experimental data are included in the published article and the Supplementary Information file.
