# Supplementary material for: Grain size dependent high-pressure elastic properties of ultrafine micro/nanocrystalline grossular
Source: Sci Rep. 2021 Nov 18;11:22481. doi: 10.1038/s41598-021-01960-6 (PMC8602367; doi:10.1038/s41598-021-01960-6)
Supplement: Supplementary file 1 — Supplementary Information. [file 41598_2021_1960_MOESM1_ESM.docx]

**Supplementary materials:**

**Grain size dependent high-pressure elastic properties of ultrafine micro**/**nanocrystalline grossular**

Jin S. Zhang^1,2*^, T. Irifune^3^, M. Hao^2^, D. Zhang^4,5^, Y. Hu^5^, S. Tkachev^4^, P. Dera^5^, J. Chen^6^, Ying-Bing Jiang^2^, Adrian J. Brearley^2^, J. D. Bass^7^, V. Prakapenka^4^

^1^ Institute of Meteoritics, University of New Mexico, Albuquerque, New Mexico 87131, U.S.A

^2^Department of Earth and Planetary Sciences, University of New Mexico, Albuquerque, New Mexico 87131, U.S.A

^3^Geodynamics Research Center (GRC), Ehime University, Matsuyama, Ehime 790-8577, Japan

^4^Center of Advanced Radiation Sources, University of Chicago, Chicago, IL 60637, USA.

^5^Hawaii Institute of Geophysics and Planetology, Department of Geology and Geophysics, School of Ocean and Earth Science and Technology, University of Hawaii at Manoa, Honolulu, Hawaii 96822, USA

^6^School of Earth and Space Sciences, University of Science and Technology of China, Hefei 230026, China

^7^Department of Geology, University of Illinois, Urbana 61801, U.S.A

^*^Email: [jinzhang@unm.edu](mailto:jinzhang@unm.edu)

1. Supplementary Figures
2. Supplementary Tables
3. **Supplementary Figures**

**
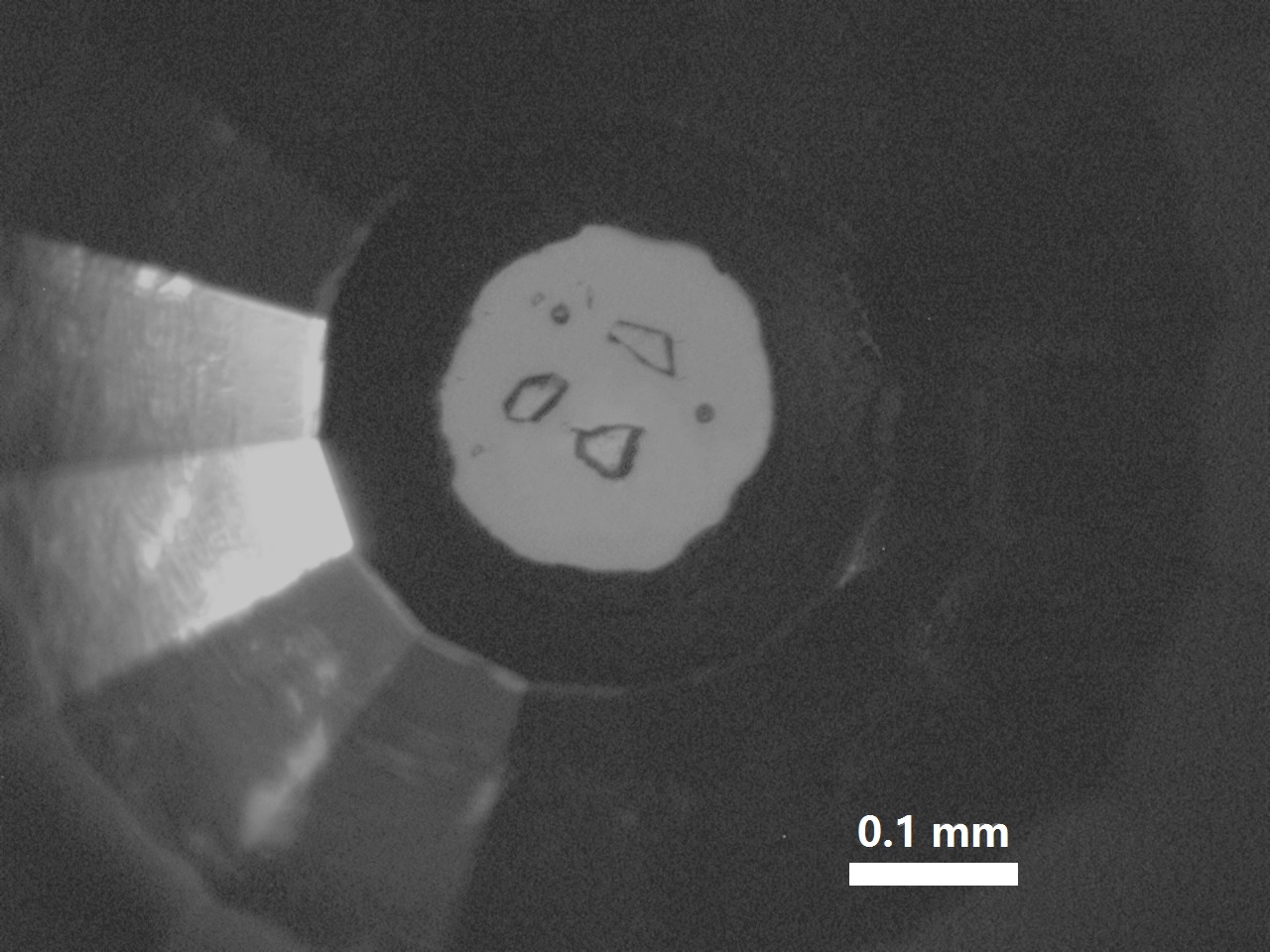
**

**Figure S1.** Garnet samples with two Ruby spheres in a diamond anvil cell for synchrotron X-ray diffraction experiments after Ne gas-loading. Samples show excellent optical quality.


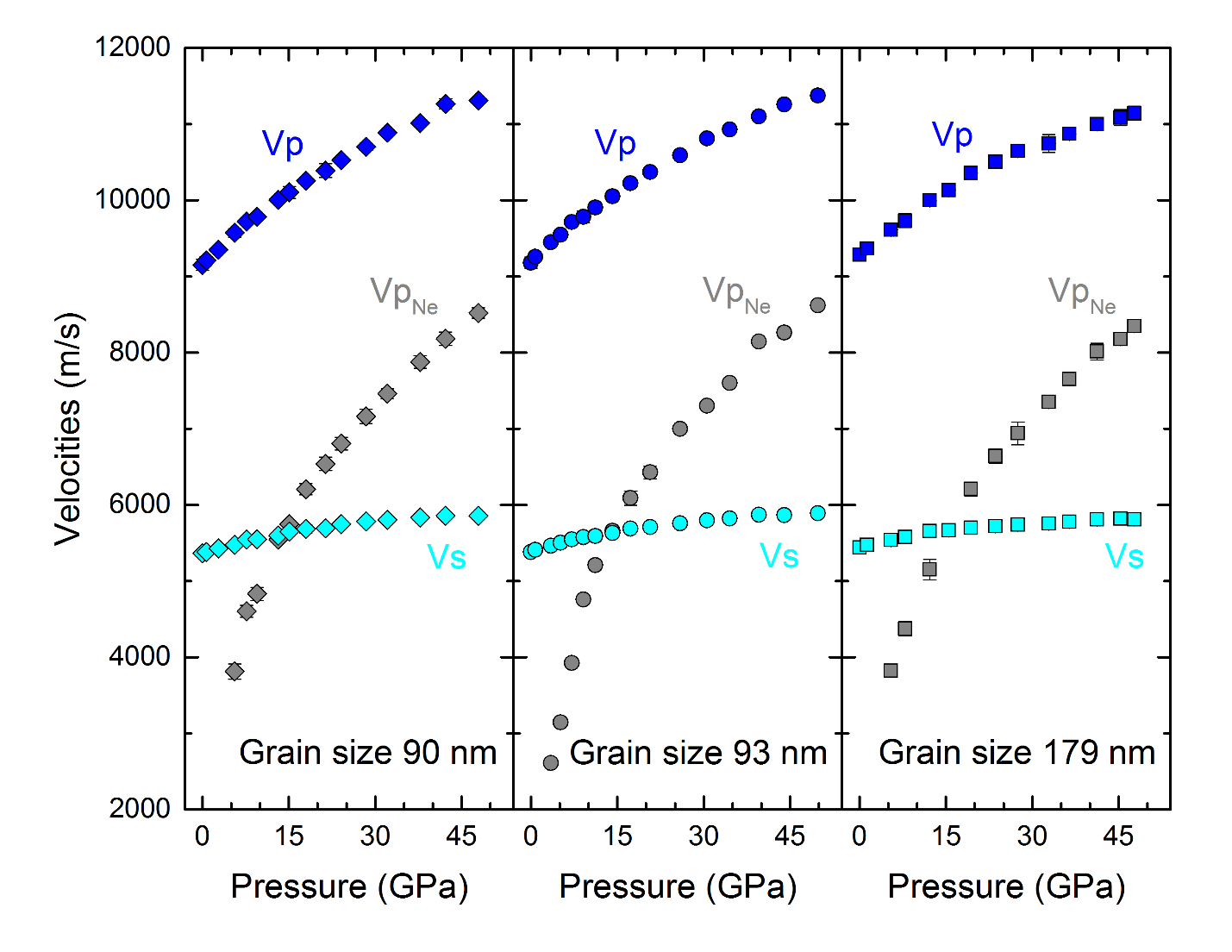


**Figure S2.** Sound velocity data for all three nanocrystalline grossular samples with Ne pressure medium. Error bars are estimated from multiple measurements at different chi angles.


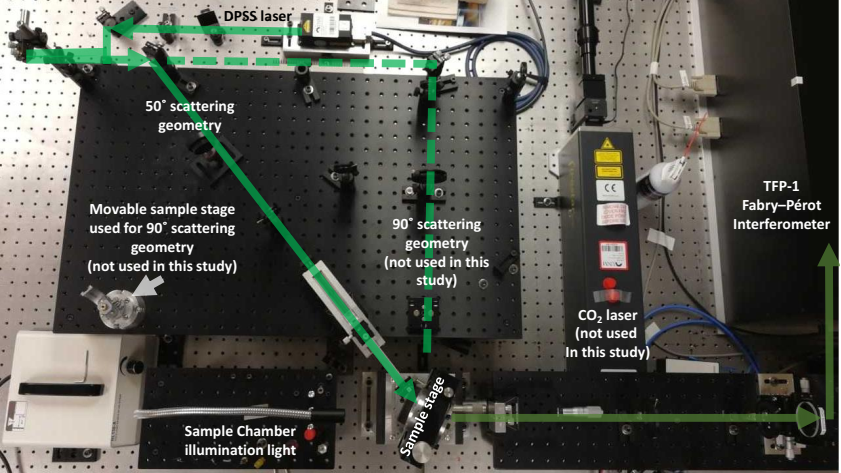


**Figure S3.** Brillouin spectroscopy system at University of New Mexico. Light paths are indicated by the green arrows.

**Figure S4.** X-Ray diffraction patterns of all 3 grossular samples at 26.3(7) GPa.


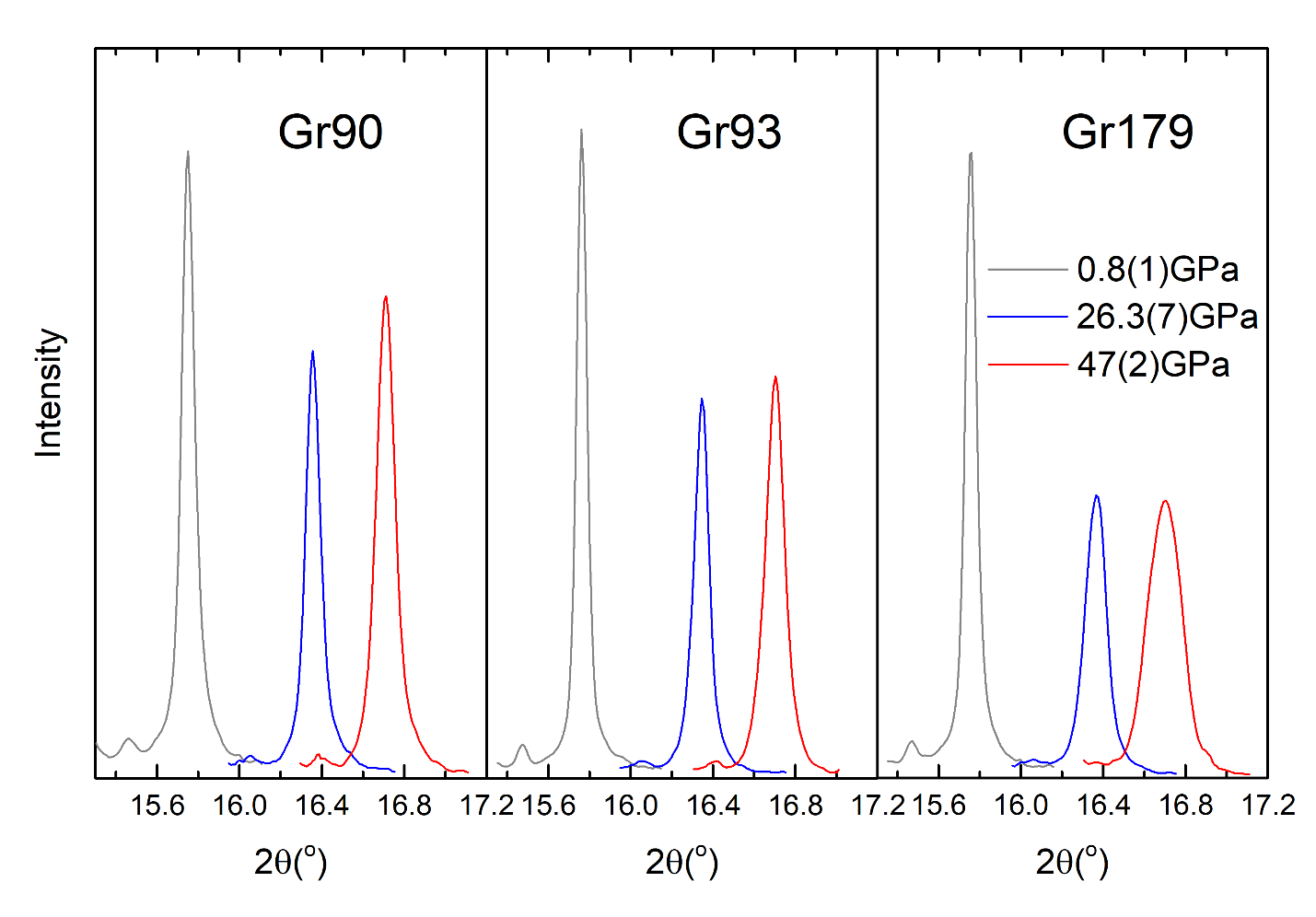


**Figure S5.** X-Ray diffraction peak (642) for all 3 nanocrystalline grossular samples at 3 different pressures. The peak clearly broadens as pressure increase.

1. **Supplementary Tables**

**Table S1.** Sound velocities of three nanocrystalline grossular samples up to 50 GPa.

| **Sample** | **Pressure (GPa)** | **Vp (km/s)** | **Vs (km/s)** | **Vp(Ne) (km/s)** |
| --- | --- | --- | --- | --- |
| Gr179 | 1 atm | 9.28(8) | 5.44(3) |  |
|  | 1.3(1) | 9.37 (6) | 5.47(4) |  |
|  | 5.5(2) | 9.61(2) | 5.54(4) | 3.82(8) |
|  | 7.9(1) | 9.73(9) | 5.58(4) | 4.4(1) |
|  | 12.2(1) | 10.00(5) | 5.65(4) | 5.1(1) |
|  | 15.5(1) | 10.13(5) | 5.67(6) | 5.67(6) |
|  | 19.3(2) | 10.36(7) | 5.70(5) | 6.21(9) |
|  | 23.6(2) | 10.50(4) | 5.72(6) | 6.64(96) |
|  | 27.5(3) | 10.65(7) | 5.74(4) | 6.9 (1) |
|  | 32.9(2) | 10.7(1) | 5.76(4) | 7.35(8) |
|  | 36.4(2) | 10.87(4) | 5.78(3) | 7.65(3) |
|  | 41.2(1) | 11.00(8) | 5.81(3) | 8.0(1) |
|  | 45.3(2) | 11.1(1) | 5.82(3) | 8.18(8) |
|  | 47.7(2) | 11.14(9) | 5.81(3) | 8.35(7) |
| Gr90 | 1 atm | 9.15(7) | 5.36(4) |  |
|  | 0.7(1) | 9.21(5) | 5.38(2) |  |
|  | 2.8(1) | 9.34(2) | 5.43(2) |  |
|  | 5.6(1) | 9.57(6) | 5.47(2) | 3.8(1) |
|  | 7.7(1) | 9.72(2) | 5.55(2) | 4.60(8) |
|  | 9.5(1) | 9.78(3) | 5.55(3) | 4.83(9) |
|  | 13.2(1) | 10.00(3) | 5.60(2) | 5.54(2) |
|  | 15.1(1) | 10.10(8) | 5.65(5) | 5.75(2) |
|  | 18.0(1) | 10.26(5) | 5.68(5) | 6.21(8) |
|  | 21.4(4) | 10.39(9) | 5.69(4) | 6.54(9) |
|  | 24.1(1) | 10.53(2) | 5.75(3) | 6.80(8) |
|  | 28.4(2) | 10.70(5) | 5.79(2) | 7.16(9) |
|  | 32.1(3) | 10.89(4) | 5.80(2) | 7.46(7) |
|  | 37.8(2) | 11.01(2) | 5.83(2) | 7.88(8) |
|  | 42.2(4) | 11.26(7) | 5.86(4) | 8.18(9) |
|  | 47.9(2) | 11.31(3) | 5.86(3) | 8.52(7) |
| Gr93 | 1atm | 9.18(4) | 5.38(4) |  |
|  | 0.8(2) | 9.26(2) | 5.41(2) |  |
|  | 3.5(2) | 9.45(2) | 5.46(2) | 2.61(2) |
|  | 5.2(1) | 9.55(6) | 5.50(3) | 3.1(1) |
|  | 7.1(1) | 9.71(7) | 5.54(2) | 3.92(2) |
|  | 9.1(2) | 9.78(2) | 5.57(2) | 4.76(2) |
|  | 11.2(1) | 9.90(5) | 5.59(5) | 5.21(2) |
|  | 14.2(2) | 10.05(4) | 5.63(4) | 5.66(2) |
|  | 17.3(1) | 10.22(2) | 5.69(2) | 6.09(2) |
|  | 20.7(1) | 10.37(2) | 5.71(2) | 6.43(9) |
|  | 25.9(1) | 10.59(3) | 5.76(2) | 7.00(2) |
|  | 30.6(1) | 10.8(1) | 5.80(4) | 7.30(3) |
|  | 34.5(1) | 10.93(6) | 5.82 (3) | 7.60(3) |
|  | 39.6(1) | 11.10(2) | 5.87(3) | 8.1(1) |
|  | 44.0(1) | 11.26(4) | 5.86(4) | 8.26(5) |
|  | 49.8(4) | 11.37(7) | 5.89(2) | 8.62(8) |

**Table S2**. Pressure-volume data of three nanocrystalline grossular samples up to 50 GPa. X-ray diffraction experiments at ambient condition were measured without diamond anvil cell and clean up slit at the experimental station 13BMC. The FWHM at ambient condition is not used in this study.

| **Pressure (GPa)** | **V (Å3)** | | |
| --- | --- | --- | --- |
|  | **Gr90** | **Gr93** | **Gr179** |
| 0 | 1670.33(2) | 1669.91(2) | 1668.22(2) |
| 0.8(1) | 1659.38(3) | 1657.28(2) | 1655.18(1) |
| 2.1(1) | 1646.79(2) | 1644.29(2) | 1642.61(3) |
| 4.2(3) | 1625.96(2) | 1623.89(2) | 1621.82(3) |
| 5.3(1) | 1618.10(5) | 1616.86(1) | 1614.79(3) |
| 7.3(7) | 1600.38(4) | 1599.97(2) | 1601.20(3) |
| 9.1(6) | 1583.61(2) | 1583.61(2) | 1585.65(4) |
| 11(1) | 1571.42(2) | 1572.63(1) | 1575.47(5) |
| 13.2(5) | 1555.25(4) | 1554.85(3) | 1555.25(2) |
| 15.9(4) | 1537.60(3) | 1538.00(3) | 1539.20(3) |
| 18.4(4) | 1523.26(3) | 1524.05(5) | 1526.04(6) |
| 20.5(3) | 1511.77(4) | 1512.56(6) | 1515.33(7) |
| 23.3(5) | 1497.19(5) | 1496.41(6) | 1500.7(1) |
| 26.3(7) | 1482.71(7) | 1480.37(8) | 1485.5(2) |
| 29.2(5) | 1467.94(7) | 1466.4(1) | 1471.0(2) |
| 32.0(4) | 1454.80(7) | 1453.65(8) | 1458.3(2) |
| 34.6(4) | 1442.51(5) | 1439.83(8) | 1445.2(3) |
| 37.1(8) | 1430.67(3) | 1426.49(8) | 1433.0(5) |
| 39.6(8) | 1417.4(1) | 1418.1 (1) | 1421.6(6) |
| 42(1) | 1407.9(1) | 1409.1(1) | 1411.3(6) |
| 44(1) | 1397.8(1) | 1400.0(1) | 1401.5(7) |
| 47(2) | 1391.05(9) | 1390.7(1) | 1392.9(8) |
| 49(1) | 1381.73(8) | 1381.7(1) | 1384.0(6) |
| 51(2) | 1373.6(1) | 1373.9(1) | 1375.8(6) |
